# Intranasal Vaccination with a Respiratory-Syncytial-Virus-Based Virus-like Particle Displaying the G Protein Conserved Region Induces Severe Weight Loss and Pathology upon Challenge with Wildtype Respiratory Syncytial Virus

**DOI:** 10.3390/v16060843

**Published:** 2024-05-24

**Authors:** Megolhubino Terhüja, Manjunath Siddappa, Pramila Lamichhane, Chetan D. Meshram, Timothy A. Snider, Jerry W. Ritchey, Antonius G. P. Oomens

**Affiliations:** 1 Department of Veterinary Pathobiology, College of Veterinary Medicine, Oklahoma State University, Stillwater, OK 74078, USAjerry.ritchey@okstate.edu (J.W.R.); 2 Department of Veterinary Sciences and Animal Husbandry, Chitradurga 577502, Karnataka, India; 3 RNA Viruses Section, Laboratory of Infectious Diseases, National Institute of Allergy, Immunology, and Infectious Diseases, NIH, Bethesda, MD 20892, USA; 4 CSIR-Central Drug Research Institute, Lucknow 226031, Uttar Pradesh, India

**Keywords:** respiratory syncytial virus (RSV), virus-like particle (VLP), vaccine, G protein, prefusion F

## Abstract

Respiratory syncytial virus (RSV) is a major cause of severe respiratory tract disease worldwide, and a pediatric vaccine is not available. We generated a filamentous RSV-based virus-like particle (VLP) that presents the central conserved region of the attachment protein G. This was achieved by co-expressing the matrix protein, phosphoprotein, nucleoprotein, and a hybrid fusion protein in which the F ectodomain was replaced with the G central region (GCR). The latter is relatively conserved and contains a receptor binding site and hence is a logical vaccine target. The immunogenicity and efficacy of the resulting VLP, termed VLP-GCR, were examined in mice using intranasal application without adjuvant. VLP-GCR induced substantial anti-N antibody levels but very low anti-G antibody levels, even after three vaccinations. In contrast, a VLP presenting prefusion-stabilized fusion (preF) protein instead of GCR induced both high anti-F and anti-nucleoprotein antibody levels, suggesting that our GCR antigen was poorly immunogenic. Challenge of VLP-GCR-vaccinated mice caused increased weight loss and lung pathology, and both VLPs induced mucus in the lungs. Thus, neither VLP is suitable as a vaccine for RSV-naive individuals. However, VLP-preF enhanced the proportion of preF antibodies and could serve as a multi-antigen mucosal booster vaccine in the RSV-experienced population.

## 1. Introduction

Respiratory syncytial virus (RSV) is a major world-wide health problem that affects all ages and can cause serious respiratory tract diseases such as bronchiolitis and pneumonia [1,2]. Recently, after decades of research, several vaccines based on purified prefusion-stabilized fusion (F) protein were approved, targeting the elderly (Abrysvo, Arexvy) and pregnant women (Abrysvo). The latter showed significant protection of newborns via passively acquired antibodies (Abs), a major accomplishment in the fight against RSV. However, a vaccine for children is not yet available. A vaccination trial in children in the 1960s using formaldehyde-treated RSV (FI-RSV) failed to protect vaccinated individuals and instead induced a virus-enhanced lung disease phenomenon (VED) upon subsequent natural RSV exposure [3,4]. Since then, several live and non-live approaches have been developed and tested, showing that it is exceedingly difficult to produce a pediatric vaccine that is both sufficiently efficacious and safe (for review see [5,6]).

A virus-like-particle (VLP) is a non-live approach that has shown promise, and in recent years, several VLP-based vaccines have been approved in several countries and shown to be effective: human papilloma virus (Gardasil; Cervarix), hepatitis-B virus (Engerix-B; Recombivax HB; Elovac B; Genevac-B; Shanvac-B), and hepatitis E virus (Hecolin). VLPs, in general, can induce relevant immune responses and can cross-present to induce both Th1 and Th2 responses [7,8,9,10]. In the case of RSV, recent reports show that VLPs can induce protective anti-RSV immunity without VED, in some cases without adjuvant [11,12,13,14,15]. The latter is true also for other non-live vaccine approaches [16,17]. These vaccines also showed long-lasting Abs relative to live RSV, presumably due to the absence of immune dysregulation by de novo synthesized RSV proteins [13].

Almost all current pre-clinical VLP approaches that target RSV use heterologous VLPs incorporating the preF protein or preF and G proteins. We previously reported the generation of authentic RSV-based VLPs [18], and here, we explore the suitability of these VLPs as vaccines. A potential advantage of RSV-based VLPs over non-RSV based VLPs is the inclusion of multiple RSV targets, including conserved core proteins, which could broaden and strengthen the immune response [19,20]. For example, humoral and T cell responses have been documented against the nucleoprotein (N) and matrix protein (M), which represent virulence factors and contribute to the dysregulation of host processes by RSV [21,22,23,24,25,26,27,28]. Furthermore, peptides derived from N, P, and M proteins were shown to induce IFN-γ production [29]. In addition to providing broader targets, an authentic RSV VLP also avoids immunity against non-target components from the heterologous packaging system.

**Figure 1:**
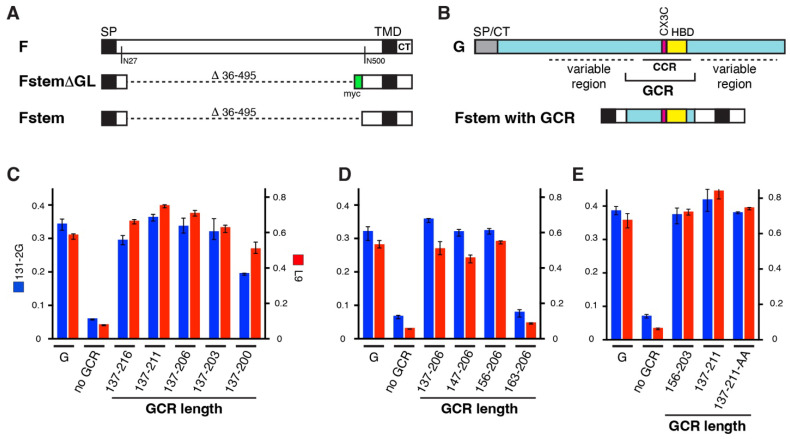
Surface expression and antibody (Ab) recognition of transiently expressed Fstem–GCR hybrid proteins. (**A**) An F construct lacking the F ectodomain (Fstem) was generated by removing the myc epitope from previously described FstemΔGL [18]. For wt fusion protein (F), only relevant N-linked glycosylation sites are shown. (**B**) Peptides (of various lengths) representing the GCR were grafted onto Fstem. SP/CT = signal peptide/cytoplasmic tail. (**C**–**E**) Hybrid Fstem GCR proteins with varying GCR lengths were transiently expressed in HEp-2 cells. At 28 hpt, cell ELISA was performed using two previously characterized anti-CCR Abs (131-2G and L9). The signals were compared to those obtained from a construct expressing wt G (G) or Fstem without a GCR graft (no GCR). (**C**) GCRs with variations at the carboxy-terminal end. (**D**) GCRs with variations at the amino-terminal end. (**E**) Based on results of (**C**,**D**), Fstem constructs containing a short and long version of the GCR (GCR156-203 and GCR137-211, respectively) were generated and similarly examined side-by-side. For GCR137-211, an additional variant was included in which the final two residues were mutated to alanine (GCR137-211-AA). (**C**–**E**) The left axis represents the OD_490_ signal from Ab 131-2G; the right axis represents the OD_490_ signal from L9. Error bars represent standard deviation of the mean of triplicate samples. The experiment was carried out twice with similar results.

Among the RSV glycoproteins, the attachment protein G and fusion protein F are the major protective antigens, and each induces neutralizing anti-viral Abs [30,31]. The F protein is essential and relatively well conserved and hence a logical target for vaccine development. Infection with RSV induces Abs against both the prefusion (preF) and post-fusion (postF) conformations [32,33], but anti-preF Abs are responsible for the majority of the neutralizing capacity in humans [33]. Anti-G antibodies also show significant potential for the prevention and treatment of RSV-associated disease (for review see [34]). G, however, is poorly conserved between viral strains, except for the signal peptide/transmembrane domain and an approximately 50-amino-acid region in the center, termed the central conserved region (CCR), which is located in between two large highly variable mucin-like regions [35,36,37] (Figure 1B). Within the CCR is a cysteine-rich site (CWAIC or CX3C) that serves as a receptor binding domain [38,39] and which was shown to mimic fractalkine, a chemokine known to be involved in leucocyte chemotaxis [40]. Interaction of CX3C and its receptor (CX3CR-1) was shown to be an important contributor to immune dysregulation [41,42,43,44,45,46]. Accordingly, Abs that block interaction of G with CX3CR-1 can neutralize A and B strains and lessen the overall disease severity by reducing Th2 cytokine responses, mucin, and G-mediated chemotaxis [46,47,48,49,50,51,52,53,54,55]. Thus, the CCR is a valuable vaccine target to prevent or reduce RSV-mediated disease, and, due to its conserved nature, anti-CCR Abs are more cross-protective than Abs directed at non-CCR areas of G [17,51,56,57]. However, in the context of full-length G, the CCR appears to be poorly immunogenic [58,59]. 

Given the importance of the CCR, targeting this domain via a vaccine may prove highly beneficial. A recent paper in which the G mucin domains were removed in a live attenuated RSV vaccine demonstrated this method to be as effective as a wildtype (wt) virus in preventing lung pathology in mice [60]. In another study, a VLP co-presenting F + G CCR was more effective at reducing lung titer and inflammation after challenge than a VLP with F + full-length G [61]. In another study, peptides corresponding to the CCR were trimerized and added to a preF nanoparticle, which yielded potent anti-F and anti-G immune responses and raised high levels of anti-G Abs in macaques and protected mice from challenge [17]. Here, we aimed to focus the immune response on the CCR by presenting it in isolation (away from the variable G regions and the F protein) in a homologous RSV-based filamentous VLP.

We previously reported that the RSV phosphoprotein (P), M, and F proteins were sufficient for the formation of wt virus-resembling filamentous VLPs. Aside from the absence of G, the P-, M-, and F-induced VLPs were indistinguishable from wt RSV via scanning electron microscopy (SEM) [18]. For F, the carboxy-terminus was required and sufficient for assembly; an F peptide representing F without the majority of its ectodomain (deletion of amino acids 36-495), termed FstemΔGL (Figure 1A), led to robust VLP formation. By grafting foreign peptides onto FstemΔGL, we were also able to incorporate peptides into filamentous particles [18]. Along with previous work [62,63], this points to the C-terminus of F as a virion incorporation signal. In this study, we took advantage of the requirement for FstemΔGL as a structural component of filamentous VLPs by grafting variations of G peptides onto FstemΔGL. The grafted peptides all contained the middle region of G, including portions or all of the G heparin-binding domain (HBD) [64], and were termed G central regions (GCR) (Figure 1B). An optimal GCR length was selected, and VLPs were generated by co-expressing this GCR with P, M, and N. Although not required for VLP formation, for the vaccine studies, N was included because N induces a significant humoral and cellular response in humans [21,22,23,24] and because recent work suggests that N is a significant virulence factor, maintaining protein translation, sequestering innate immunity components [65,66,67], and interfering with the immune synapse [25]. A VLP displaying the GCR was tested for its ability to induce an immune response in mice. Vaccines were applied intranasally (IN) because recent papers show an important contribution of local mucosal humoral and cellular immunity in countering virus infection, replication, and disease severity via induction of mucosal IgA and resident memory and effector T cells [29,68,69,70,71]. No adjuvant was used due to safety concerns with some IN-applied adjuvants [72,73,74,75] and because several previous RSV studies have successfully induced immunity without an adjuvant [13,14,16,17].

Here, we tested the capacity of VLP-GCR to induce a humoral immune response after IN application. To better assess immunogenicity, we also compared it to VLPs expressing the preF protein instead of G. Neither VLP protected mice from challenge virus-induced disease, and VLP-GCR induced severe weight loss and lung pathology. Neither VLP, therefore, constitutes an appropriate stand-alone vaccine for RSV-naive individuals. However, as VED does not occur in RSV-experienced individuals [76], and due to its broad immunogenicity and potential to focus the anti-F response on prefusion F, VLP-preF could be a suitable booster vaccine for the RSV-experienced population.

## 2. Materials and Methods

### 2.1. Cells, Antibodies, and Viruses

HEp-2 cells (source: American Type Culture Collection) were maintained in advanced Dulbecco’s modified Eagle medium (DMEM) supplemented with 4% fetal bovine serum, 50 units/mL penicillin, 50 μg/mL streptomycin, and 2 mM glutamax (Invitrogen, Waltham, MA, USA). HEK-293 Freestyle cells were maintained in Freestyle 293 Expression medium (Invitrogen, Waltham, MA, USA). Mab L9 was provided by Ed Walsh (University of Rochester School of Medicine, Rochester, NY, USA) [77]. MAb 131-2G was provided by Lia Haynes (CDC) and Larry Anderson (Emory University, Atlanta, GA, USA) [78]. Anti-flag antibody was acquired from Genscript (Piscataway, NJ, USA. Anti-N antibody was acquired from AbD serotec. To detect M, a previously described rabbit polyclonal anti-M peptide serum was used [79]. D25 and motavizumab were kindly provided by Jason McLellan (University of Texas at Austin, Austin, TX, USA). The wt virus used was a fully wt A2 strain of RSV that was recovered from cDNA, as previously described [32].

### 2.2. Plasmids

Plasmids expressing codon-optimized versions of N, P, and M were previously described [18,32]. P contained a C-terminal flag tag (DYKDDDDL) to facilitate detection via Western blots, previously described as Pflag240 [80]. To produce VLP-preF, a flag-tagged N was used, in which the flag tag was inserted at position 3. Hybrid protein Fstem/GCR was constructed from a previously described F protein, lacking its ectodomain (amino acids 36-495) (FstemDGL) [18]. To generate Fstem, a myc tag present in FstemDGL was deleted, and F amino acids 573 and 574 were mutated to alanines via site-directed mutagenesis. Different lengths of the GCR were PCR-amplified and cloned into Fstem using conventional cloning techniques. A construct containing G amino acids 137-211, and with F amino acids 573 and 574 mutated to alanines, was selected for mouse experiments and termed Fstem/GCR. The preF plasmid was based on a previously reported codon-optimized F open reading frame [62], in which four stabilizing DS-Cav1 mutations [81] were introduced via site-directed mutagenesis [32]. In addition, amino acids 573 and 574 were mutated to alanines.

### 2.3. Cell ELISA to Measure Fstem/GCR Levels on the Surface of Transfected Cells (Figure 1)

Relative surface levels of Fstem GCR hybrid proteins were determined by cell ELISA, as previously described [18,32], with minor modifications. In short, HEp2 cells were transfected with Fstem plasmids containing GCR peptides of different lengths. As controls, plasmids expressing wt G protein or Fstem without GCR were included. At 28 hpt, cells were incubated for 2 h with anti-G Abs L9 or 131-2G, washed to remove unbound Abs, and then briefly fixed with 4% freshly dissolved paraformaldehyde. Primary Ab incubation prior to fixation avoids potential structural or conformational changes to the GCR peptide induced by fixation. This was followed by incubation with a horseradish peroxide-conjugated (HRP) secondary antibody and washing steps. Finally, cells were incubated in O-phenylenediamine dihydrochloride (OPD) (Thermo Fisher Scientific, Waltham, MA, USA)-based ELISA substrate, and the reaction was stopped by adding to 3M sulfuric acid. The optical density at 490 nm (OD_490_) was measured in a Versamax plate reader (Molecular Devices, San Jose, CA, USA). The reported values epresent the average and standard deviations of one experiment with triplicate samples. The experiment was carried out twice with similar results. 

### 2.4. Western Blots (Figure 2)

**Figure 2:**
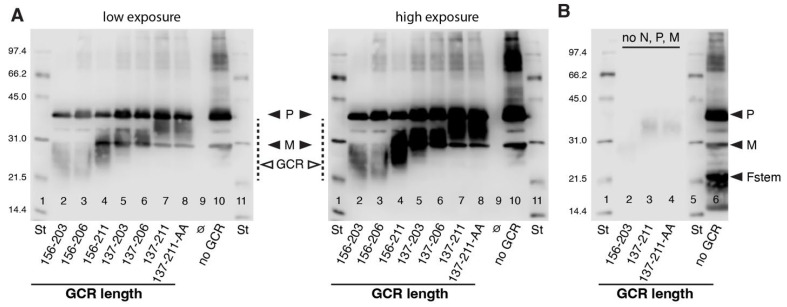
Generation of RSV VLPs carrying Fstem hybrid proteins with GCR peptides of various lengths. Fstem GCR hybrid proteins were coexpressed with N, P, and M in HEp-2 cells, and VLPs were harvested at 44 hpt, as described in the text. Semi-purified VLPs were mixed with Laemmli buffer and electrophoresed, and Western blots were generated using anti-flag Ab (to detect P), anti-M Abs [79], and anti-G Ab 131-2G. An anti-N Ab was not available and not included. (**A**) VLPs with GCR peptides of different lengths. Two different exposures are shown—No GCR: VLPs that lack a GCR (co-expression of P, M, and FstemΔGL); St: protein standard. On the left, molecular weights are indicated. ∅ indicates an empty lane. (**B**) Hybrid proteins with GCR156-203, 137-211, and 137-211-AA were expressed in the absence of P and M (lanes 2–4). Lane 6 represents co-expression of P, M, and FstemΔGL, in which the FstemΔGL was visualized by addition of an anti-myc Ab.

Plasmids expressing Fstem hybrid proteins with GCR peptides of various length were co-transfected with plasmids encoding N, flag-tagged P, and M into HEp-2 cells (4 h). As controls, a plasmid expressing Fstem without GCR was included. In Figure 2B, plasmids expressing Fstem GCR hybrid proteins were transfected in the absence of N, P, and M. At 22 h after the start of transfection, the medium was replaced with OPTIMEM without additives. VLPs were harvested at 44 hpt, as described in the text. Equal volumes of semi-purified VLPs were mixed 1:1 with 2× Laemmli buffer, electrophoresed (12% gel, reducing), and transferred to Immobilon membrane using a semidry apparatus (Bio-Rad, Hercules, CA, USA). Blots were probed with anti-flag Ab (to detect P), anti-M Abs [79], and anti-G Ab 131-2G. An anti-N Ab was not available and not included. In Figure 2B, Fstem was visualized by including an anti-myc Ab during primary Ab incubation. After incubation with HRP-conjugated secondary Ab, blots were developed using enhanced chemiluminescence and scanned on a C-DiGit scanner (LI-COR Biosciences, Lincoln, NE, USA). The experiment was carried out twice and two different exposures of a representative blot are shown.

### 2.5. Western Blots (Figure 4 and Figure 6)

Semi-purified VLP-GCR and VLP-preF were pelleted at high g-force (20,000× *g*, 60 min) and resuspended in 1× Laemmli buffer and boiled. A lysate of HEp2 cells infected with wt RSV was used as positive control, and an untransfected 293 or HEp-2 cell lysate was used as negative control. The samples were electrophoresed, transferred, and processed as described above, except that blots were scanned on an Amersham Imager 600 (General Electric, Boston, MA, USA). The blots were probed with 131-2G Ab (G) or Motavizumab (F), anti-M, and flag Ab (to detect flag-tagged P and N). The experiment was carried out twice, and a representative blot is shown.

### 2.6. Electron Microscopy (Figure 3)

**Figure 3:**
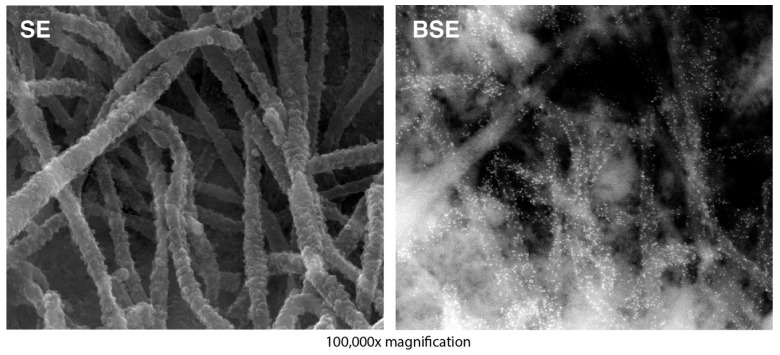
High-resolution analysis of VLP-GCR. HEp-2 cells grown on plastic coverslips were transfected with plasmids expressing N, P, M, and a hybrid Fstem protein containing GCR137-211-AA. At 26 hpt, the GCR was labeled with Ab L9, followed by a secondary Ab conjugated to 10 nm gold, washed, and processed for SEM. Samples were examined using secondary electron (SE) (**left panel**) and backscattered electron (BSE) (**right panel**) modes and photographed at 100,000× magnification. Gold-labeled GCR is visible as white dots.

HEp-2 cells on plastic coverslips were co-transfected with plasmids expressing N, flag-tagged P, M and Fstem/GCR. At 26 hpt, cells were fixed with 4% freshly dissolved paraformaldehyde for 15 min. Fixed cells were incubated with anti-G antibody (L9). Cells were washed and incubated with goat-anti-mouse Abs conjugated to 10 nm colloidal gold (Aurion). Cells were washed three times and fixed in 2.5% glutaraldehyde for 120 min at room temperature. After fixation, cells were washed once, incubated in 1% osmium tetroxide for 1 h, and washed three additional times. Cells were then dehydrated in ethanol in a stepwise fashion and incubated in hexamethyldisilazane for 1 min. Following hexamethyldisilazane treatment, samples were air-dried, carbon-coated, and examined in an FEI Quanta 600 field-emission gun scanning electron microscope. Samples were examined using secondary electron (SE) and backscattered electron (BSE) modes and photographed at 100,000×. For images shown in Figure 3, contrast and brightness were slightly adjusted with Photoshop to better visualize the gold particles.

### 2.7. Production of VLPs for Mouse Studies

VLP-GCR was produced by co-transfecting plasmids encoding flag-tagged N, Pflag, M, and Fstem/GCR137-211-AA into 293 Freestyle cells (Invitrogen, Waltham, MA, USA) in suspension. The transfection solution was replaced with Freestyle medium after 5 h. At 22 hpt, Freestyle medium was replaced with OPTIMEM (Invitrogen, Waltham, MA, USA) without additives. At 44 hpt, cells were pipetted up and down for 2 min, and cells and cell debris were removed by low-speed centrifugation. VLPs were pelleted through a 25% sucrose cushion by ultracentrifugation at 10,000× *g* for 65 min at 4 °C. The pellets were washed once, resuspended in cold PBS, flash frozen, and stored at −80 °C. The total protein concentration in the VLPs was determined using a Micro BCA Protein Assay Kit (Thermo Fisher Scientific, Waltham, MA, USA). VLP-preF were generated as described above, with the exception that a preF expressing plasmid was used in place of an Fstem/GCR expressing plasmid.

### 2.8. Vaccination Protocol and Serum Ab Collection (Figure 4 and Figure 6)

Groups of female BALB/c mice (eight-week-old, *n* = 5 per group) were anesthetized by intraperitoneal injection with 100 mg/kg of body weight of ketamine and 5 mg/kg of xylazine. Mice received three vaccinations (prime on day 0; boost 1 on day 14 after prime; boost 2 on day 35 after prime) using the IN application (50 µL volume). The VLP amounts ranged from 25 to 100 μg, as indicated in the results section. The mock vaccine group received 50 μL of PBS. As a positive control, one group of mice was similarly vaccinated with wt RSV (500,000 PFU in a 50 μL volume). At 21 days post-2nd boost, blood samples were collected for serum Ab studies using ELISA (see below).

### 2.9. ELISA to Measure Anti-Viral Ab Levels in Mouse Sera (Figure 4 and Figure 6)

Anti-viral IgG Abs in mouse sera were determined via ELISA. Nickel-coated plates were incubated overnight with his-tagged purified G protein (Sino Biological, Houston TX, USA), preF or postF proteins (gifts from Jason McLellan), or N protein (Sino Biological, Houston, TX, USA). Coated plates were washed, blocked, and incubated (2 h, room temperature) with individual mouse sera diluted in 3-fold steps, with a starting dilution of 1:100. As an additional negative control, commercially acquired mouse IgG isotype Abs were included. After primary Ab incubation, plates were processed and developed as described above for cell ELISA. Note that the G, preF, and postF antigens and ELISAs were validated in previous work [32,82]. The N antigen and anti-N Ab ELISA were validated in Figure S4. Different amounts of his-tagged N were used for coating. Plates were washed, blocked, and incubated with sera from wt RSV-infected mice as well as a commercially available anti-N Ab (AbD serotec). Wells without N coating were used as a negative control.

### 2.10. Challenge Studies (Histopathology) (Figure 5 and Figure 7)

For challenge studies, mice were vaccinated or mock-vaccinated (as a negative control for protection) as described above and challenged with two million PFU of wt RSV at four weeks post-2nd boost. Five days post-challenge, mice were humanely sacrificed, and lungs were collected for histopathology analysis. Lungs were fixed in 10% neutral-buffered formalin and then routinely processed through graded alcohols and xylene, embedded in paraffin, sectioned at 4 μm, and stained with hematoxylin and eosin (H&E) (Sigma, St. Louis, MO, USA. Slides were scored for parameters assessed for RSV-induced pathology, scoring from 0 (no lesions) to 3 (marked lesions), attributed to perivascular cuffing (PVC; leukocytes surrounding blood vessels), peribronchiolar cuffing (space and fluid surrounding bronchioles), interstitial pneumonia (thickness of alveolar septa; leucocytes in the alveolar space), edema (flooding of lymph vessels surrounding blood vessels and airways), mucus, eosinophil influx, and neutrophil influx [32,82]. Lesion scoring was performed by board-certified (ACVP) veterinary pathologists blinded to study groups. The average total pathology scores were determined for each group (*n* = 5) by adding up all parameter scores from individual mice and then dividing by five (note that in Figure 7, to save animals, only 3 mice/group were used for control groups RSV wt and mock).

### 2.11. Mouse Ethics Statement

All mouse studies were approved by the Institutional Biosafety Committee and Institutional Animal Care and Use Committee at Oklahoma State University. Female BALB/c mice were purchased from Jackson Laboratory (Bar Harbor, ME, USA). The experiments were performed in strict accordance with the Office of Laboratory Animal Welfare guidelines and the Public Health Service Policy on Human Care and Use of Laboratory Animals. Euthanasia was consistent with the guidelines of the American Veterinary Medical Association.

### 2.12. Statistics

**Figure 4:**
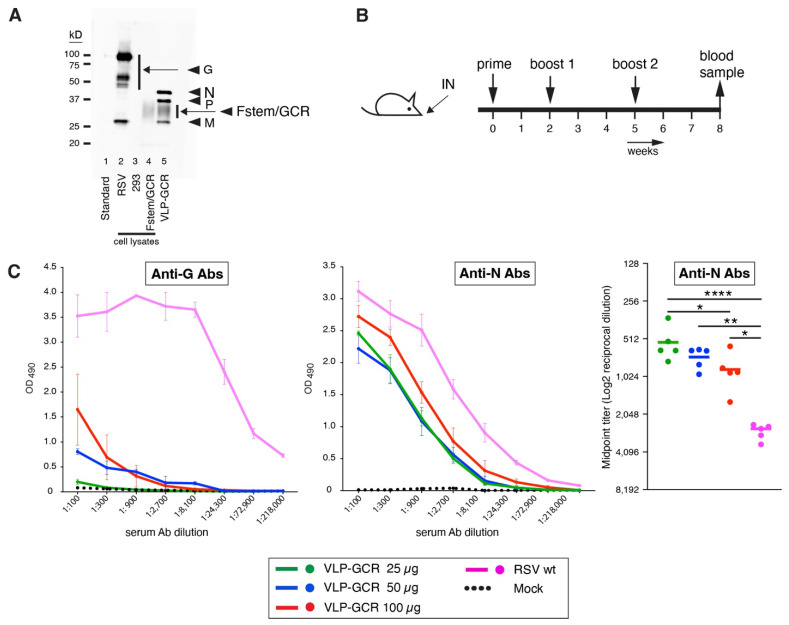
Antiviral Abs induced after vaccination with VLP-GCR. (**A**) VLP-GCR were generated by co-expressing flag-tagged N and P, M, and Fstem/GCR137-211-AA in suspension with 293 cells and harvested at 44 hpt, as described in Section 3.4 and Materials and Methods. Semi-purified VLPs were mixed with Laemmli buffer and electrophoresed, and Western blots were generated using anti-flag Abs (to detect P and N), anti-M serum [79], and anti-G Ab L9. The equivalent of 8 µg VLP-GCR was loaded based on total protein as determined via BCA assay. As controls, the following were included: a cell lysate of RSV-infected HEp-2 cells (RSV); a cell lysate of 293 cells (293); and a cell lysate of Fstem/GCR-transfected 293 cells (Fstem/GCR). Standard: protein ladder. On the left, molecular weights are indicated (kD). (**B**) eight-week-old BALB/c mice were thrice vaccinated IN without adjuvant, as shown, using 25, 50, or 100 µg of VLP-GCR. As a comparison, mice were similarly vaccinated with wt RSV or mock-vaccinated. Three weeks after the final vaccination, blood samples were taken, and serum Ab levels were examined via ELISA, using commercially acquired purified G or N proteins to coat the plates. (**C**) Anti-G and anti-N Ab levels. Mock: Animals thrice vaccinated with PBS. Error bars indicate standard deviations from the means of 5 individual mice. From the Ab curves, midpoint titers were derived using non-linear regression analyses. Horizontal bars indicate the mean of 5 individual mice. Asterisks indicate significant differences (* *p* < 0.05, ** *p* < 0.01, **** *p* < 0.0001). Due to low Ab levels, anti-G titers could not be quantitated.

**Figure 5:**
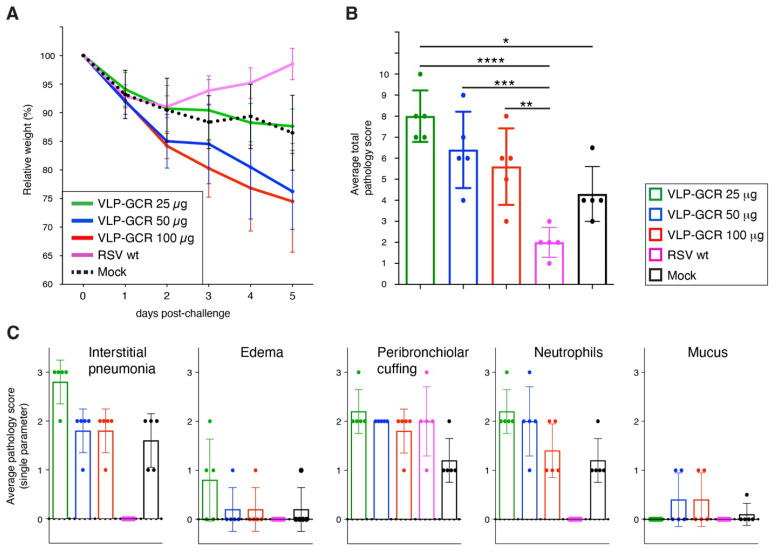
Weight loss and lung pathology after challenge of VLP-GCR vaccinated mice with wt RSV. Mice were vaccinated with VLP-GCR, as shown in Figure 4B, and challenged with two million PFU of wt RSV four weeks after the 2nd boost. (**A**) Relative weights post-challenge. (**B**,**C**) Lung pathology after challenge. Five days post-challenge, lungs from challenged mice were collected and processed for histological examination using H&E stain. Tissues were examined and scored blindly by an ACVP-certified veterinary pathologist using five distinct disease parameters (see text), scoring each parameter from 0 (no pathology) to 3 (high pathology). Mock = PBS-vaccinated mice. Bars represent the average total pathology score (**B**) or average single pathology parameter score (**C**). Error bars represent the standard deviation of the mean of five individual mice. For (**B**), statistical differences were determined via one-way ANOVA (* *p* < 0.05, ** *p* < 0.01, *** *p* < 0.001, ****, *p* < 0.0001).

**Figure 6:**
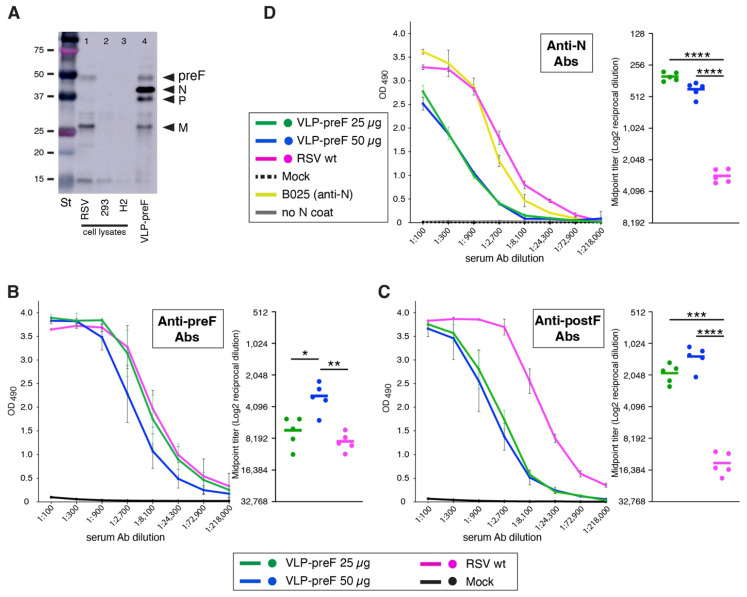
Serum Ab levels induced by a VLP expressing preF. (**A**) VLPs carrying preF protein (VLP-preF) were generated by co-expressing flag-tagged N and P, M, and preF in suspension with 293 cells and harvested at 44 hpt, as described in Section 3.4 and Materials and Methods. Semi-purified VLPs were mixed with Laemmli buffer and electrophoresed, and Western blots were generated using anti-flag Abs (to detect P and N), anti-M serum [79], and anti-F Ab motavizumab. The equivalent of 8 µg VLP-preF was loaded based on total protein as determined via BCA assay. As controls, cell lysates of 293 cells (293) and RSV-infected HEp-2 cells (RSV) were included. Lane 3 is a lysate of uninfected HEp-2 cells (H2) as an additional negative control. St: protein standard. On the left, molecular weights are indicated (kD). (**B**–**D**) Eight-week old BALB/c mice were thrice vaccinated IN without adjuvant, with 25 or 50 μg of VLP-preF, as shown in Figure 4B. As a comparison, mice were thrice vaccinated three weeks apart with wt RSV, or thrice mock-vaccinated (PBS). Three weeks post-vaccination, blood samples were taken, and serum Ab levels were examined via ELISA, using purified preF, postF, or N proteins to coat the plates. (**B**) Anti-preF Ab levels. (**C**) Anti-postF Ab levels. (**D**) Anti-N Ab levels. Error bars indicate standard deviations from the mean of 5 individual mice. From the Ab curves, midpoint titers were derived by non-linear regression analysis. Horizontal bars indicate the mean of 5 individual mice. Asterisks indicate significant differences (* *p* < 0.05, ** *p* < 0.01, *** *p* < 0.001, ****, *p* < 0.0001).

**Figure 7:**
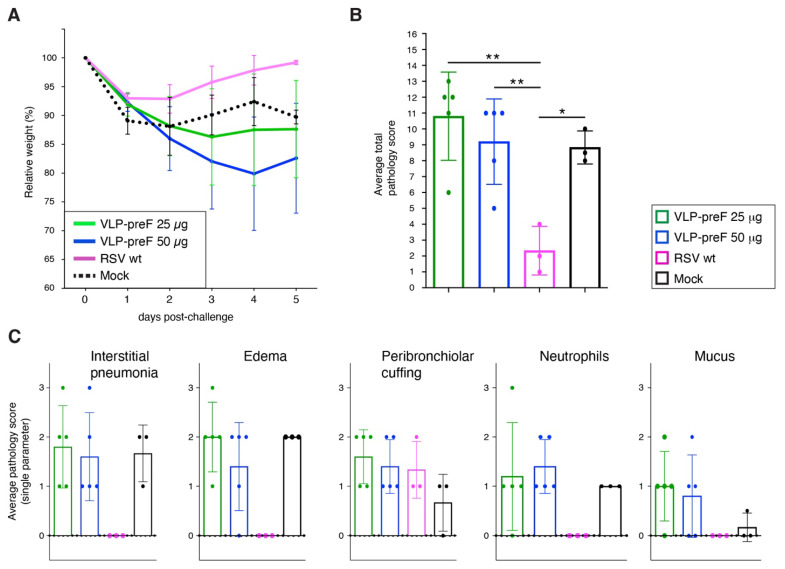
Weight loss and lung pathology after challenge of VLP-preF vaccinated mice with wt RSV. Mice were vaccinated with VLP-preF in a regimen, as shown in Figure 4B, and challenged with two million PFU of wt RSV four weeks after the final boost. (**A**) Relative weights post-challenge. (**B**,**C**) Lung pathology after challenge. Five days post-challenge, lungs from challenged mice were collected and processed for histological examination using H&E stain. Tissues were examined and scored blindly by an ACVP-certified veterinary pathologist using five distinct disease parameters (see text), scoring each parameter from 0 (no lesions) to 3 (marked lesions). Mock = PBS-vaccinated mice. Bars represent the average total pathology score (**B**) or average single pathology parameter score (**C**). Error bars represent the standard deviation of the mean of five individual mice (except for RSV and mock, 3 mice/group were used). For (**B**), statistical differences were determined by one-way ANOVA (* *p* < 0.05, ** *p* < 0.01).

All statistics were performed using Graphpad Prism 10. For Figure 4 and Figure 6, midpoint titers were derived from the Ab curves via non-linear regression using Graphpad Prism 10. When Ab levels were very low, midpoint titers could not be determined. For Figure 4, Figure 5, Figure 6 and Figure 7, means represent five individual mice, and significant differences between vaccines were determined via one-way ANOVA with Tukey’s post hoc test (* *p* < 0.05, ** *p* < 0.01, *** *p* < 0.001, ****, *p* < 0.0001; ns, not significant). 

## 3. Results

### 3.1. Determining the GCR Length for Optimal Surface Expression and Recognition by Known Anti-CCR Abs (Figure 1)

We previously reported the design of FstemΔGL (Figure 1A), an F construct that lacked amino acids 36-495 (ectodomain) and was sufficient to induce formation of RSV-resembling filamentous VLPs in the presence of P and M (see Introduction) [18]. In the same work, we found that unrelated proteins could be incorporated into VLPs by grafting onto Fstem∆GL. The latter contained a myc tag and also lacked the remaining native F glycosylation sites at asn residues 27 and 500. For this project, the myc tag of Fstem∆GL was removed, and the resulting base construct was designated Fstem (Figure 1A). To achieve a construct with strong surface expression, GCR regions of different lengths were grafted onto Fstem (Figure 1B), and the resulting constructs were transfected into HEp-2 cells and examined for surface expression by cell ELISA at 28 hpt (Figure 1C–E). Two known anti-CCR Abs, 131-2G [78] and L9 [77], were incubated on non-fixed unpermeabilized transfected cells to simultaneously measure surface expression and structural integrity.

First, we varied the carboxy-terminal end of the GCR, keeping the amino-terminal end constant (Figure 1C). Five different GCR constructs were made, containing a range of G residues from 137–216 to 137–200. A plasmid expressing the intact G protein was used as a positive control. All Fstem and GCR hybrid constructs were detected at levels similar to wt G. However, shortening of the GCR beyond residue 203 lowered the surface signal for both anti-CCR Abs and thus negatively impacted the surface level or the integrity/recognition of the GCR. Next, we varied the amino-terminal end of the GCR while keeping the carboxy-terminal end constant (Figure 1D). Fstem constructs containing GCR peptides starting with G residues 137, 146, and 156 yielded high signals, whereas a GCR starting with residue 163 was poorly recognized. Thus, deletion of residues past 156 also negatively impacted the surface level or the integrity/recognition. Based on the results of Figure 1C,D, we estimate that the shortest and longest GCR regions to be effectively presented by Fstem and recognized at the cell surface in the native (unfixed) state would be residues156-203 and 137-211. In Figure 1E, we directly compare Fstem hybrid proteins with GCR156-203 and GCR137-211. For hybrids with GCR137-211, we also analyzed a modification in which the two very carboxy-terminal amino acids of Fstem (native F amino acids S573 and N574) were mutated to alanines (GCR137-211-AA). In previous unpublished observations, this modification appeared to enhance the amount of virus-induced filaments at the cell surface, indicative of increased particle formation or stability. Most Fstem–GCR hybrid proteins were readily recognized by both L9 and 131-2G, suggesting that the GCR peptides are likely conformationally similar to the CCR region of wt G protein.

### 3.2. Generation of RSV-Based VLPs Containing Fstem Proteins with GCR Peptides of Various Length (Figure 2)

Figure 1 examines the cell surface expression and recognition of Fstem–GCR hybrid proteins via known anti-CCR Abs. Here, we generated VLPs by co-transfecting the various Fstem GCR hybrid proteins with N, P, and M to determine whether they incorporate into VLPs. Seven hybrid constructs were examined, using a previously established protocol for induction and harvest of VLPs [18]. Though not required for VLP formation, N was included as it was shown to be a virulence factor and to play a role in the anti-viral response (see Introduction). For P, we substituted valine 43 and serine 54 with alanines (V43A, S54A), as previous work suggests that the unphosphorylated state of these residues favors the assembly process [80]. The P protein was also tagged with a flag epitope at its carboxy-terminus (position 240 [80]) in order to detect P on Western blots.

Plasmids expressing the different Fstem GCR hybrid proteins were co-transfected with N-, P-, and M-expressing plasmids into HEp-2 cells. As a negative control for G, a co-transfection of N, P, M, and FstemΔGL (which lacks a GCR (see Figure 1A)) was included. To harvest VLPs, cells were scraped at 44 hpt and agitated by pipetting up and down. Cell debris was cleared via low-speed centrifugation, and VLPs were pelleted through a 25% sucrose cushion and dissolved in Laemmli buffer. Samples were electrophoresed, and Western blots were generated. Blots were incubated with anti-flag Ab to detect P, anti-M peptide serum [79], and anti-G Ab 131-2G (Figure 2A). As additional controls for VLP formation, Fstem constructs containing the minimum (156–203) and maximum (137–211) G sequences were also expressed in the absence of N, P, and M (Figure 2B, lanes 2–4) and compared to co-expression of N, P, M, and FstemΔGL (Figure 2B, lane 6). Expression of Fstem GCR hybrid proteins alone did not yield significant levels of VLPs on Western blots (Figure 2B), consistent with our previous finding that P, M, and F are each required for efficient particle formation. In contrast, all combinations of co-expressed viral proteins yielded VLPs that contained P, M, and Fstem GCR hybrid proteins. The hybrid proteins varied in molecular weight and were heterogenous in size, which may, in part, be due to a varied serine/threonine content with potential for O-linked glycosylation (wt G protein is highly O-link glycosylated in its variable regions (see Figure 1B)). Due to the smeary nature of the Fstem GCR hybrids, accurate quantitation could not be performed. However, the combination of N, P, M, and Fstem with GCR137-211/AA was consistently among the highest VLP yields. For this reason, and because a longer GCR peptide may enhance immunogenicity, we selected the VLP displaying GCR137-211-AA—henceforth referred to as VLP-GCR—for further characterization. Note that GCR137-211 includes the previously defined HBD [64].

### 3.3. High Resolution Analysis of VLPs Displaying GCR (Figure 3)

To examine the morphology of VLP-GCR, we performed field emission SEM on HEp-2 cells co-expressing N, P, M, and the hybrid Fstem protein displaying GCR137-211-AA. Characterization of VLPs at the cell surface is possible because, just like wt RSV, VLPs remain largely associated with the cell surface. Transfected HEp-2 cells were processed at 26 hpt. Using Ab L9, the G protein was labeled with 10 nm gold particles prior to processing for SEM, as described previously [18]. As shown in Figure 3 (left panel), transfected cells carried large numbers of filamentous particles at the cell surface. Since Fstem is sufficient for the formation of VLPs when co-expressed with P and M, the filamentous particles in Figure 3 looked very similar to those observed after P, M, and full-length F co-expression in previous work [18]. 

Backscatter electron scanning allows for the visualization of gold particles in the same sample and showed that most of the filamentous particles contained high levels of GCR Figure 3 (shown as white dots in the right panel). As a negative control, a non-relevant primary Ab was used (AcV5, [83]) (see Figure S1). Since we used a known neutralizing anti-CCR Ab (L9), the results also suggest that the GCR structure is relatively preserved and accessible to Abs.

### 3.4. IN Applied VLP-GCR Is Immunogenic but Induces Very Low Anti-G Ab Levels (Figure 4)

We examined the immunogenicity of VLP-GCR. To produce higher yields of VLP-GCR, we switched to a suspension-based shaking cell culture system, using 293 Freestyle cells and serum-free medium, as described in Materials and Methods. In addition to P, we also added a flag tag to the N protein N-terminus. VLP-GCR was semi-purified, total protein concentration was determined, and viral antigen presence was verified via Western blotting (Figure 4A). Eight-week-old BALB/c mice then received three IN vaccinations (days 0, 14, and 35) (Figure 4B), using 15, 25, or 35 µg of VLP-GCR per vaccination (Figure S2). Vaccines were applied without adjuvant because we ultimately aim to achieve a mucosal vaccine that does not require adjuvants and because other groups have found that adjuvants are not necessary for VLPs to induce a protective response [13,14]. Three weeks after the second boost (day 56), serum samples were taken, and serum Abs was measured via ELISA. This was conducted as previously described [32] but using a different commercial source for the purified G protein. Sera from VLP-GCR-vaccinated mice were compared to sera from mice identically vaccinated with wt RSV. As a negative control, animals were thrice IN mock-vaccinated (PBS). Our initial finding showed very low levels of induced anti-G Abs in VLP-GCR-vaccinated mice (Figure S2). This was true even at the highest vaccine amount (35 µg). We therefore repeated the experiment with higher amounts of VLP-GCR (25, 50, or 100 µg) (Figure 4C). We found that vaccination with VLP-GCR was well-tolerated, and significant weight loss occurred only upon wt RSV vaccination (Figure S3). Minor weight losses were seen in all groups, including mock-vaccinated mice, which is, in part, caused by the effects of ketamine/xylazine anesthesia. In the wt RSV group, there was additional (delayed) weight loss around day 6–7, which has been observed by many other groups and which is assumed to occur, in part, due to an influx of T cells into the lung [84]. Surprisingly, in spite of high doses and high levels of GCR in VLP-GCR particles, as seen by SEM and Western blotting, VLP-GCR induced very low levels of anti-G Abs compared to wt RSV (Figure 4C). Since we included N in the VLP-GCR vaccine, we similarly tested the presence of anti-N Abs. An N ELISA was validated in Figure S4. In contrast to anti-G Abs, VLP-GCR induced moderate-to-high levels of anti-N Abs (though in all cases, the levels were significantly lower than those observed after vaccination with wt RSV) (Figure 4C). Even the lowest vaccine amount (25 µg) induced substantial amounts of anti-N Abs, indicating that VLP-GCR was immunogenic after IN vaccination without adjuvant, and thereby also suggesting that the GCR itself is poorly immunogenic.

### 3.5. VLP-GCR Vaccination Induces Severe Weight Loss after Challenge with wt RSV and Does Not Protect from Lung Pathology (Figure 5)

The groups of mice vaccinated in Figure 4 were challenged with 2 million PFU of wt RSV four weeks after the second boost. Mice were weighed daily and were sacrificed on day 5 post-challenge to examine lung pathology. For the latter, lungs were processed for H&E staining and scored blindly by an ACVP-certified pathologist using the following parameters: interstitial pneumonia (thickness of alveolar septa; leucocytes in the alveolar space), edema (flooding of lymph vessels surrounding blood vessels and airways), peri-bronchiolar cuffing (space and fluid surrounding bronchioles), eosinophils, neutrophils, and mucus, scoring each parameter from 0 (no pathology) to 3 (high pathology). Note that eosinophils were not included in the scores as we and others found previously that they do not appear to be specific to RSV disease and are not a cause of enhanced disease [32].

In terms of weight loss, the only group that rapidly re-gained its initial (day 0 post-challenge) weight was the wt RSV-vaccinated group (Figure 5A). The 25 µg VLP-GCR group showed moderate weight, loss similar to that of mock-vaccinated mice. In contrast, the 50 and 100 µg VLP-GCR groups showed more severe weight loss (~25% of initial weight), and animals were still losing weight at the time of sacrifice. Pathology in the lungs of mock-vaccinated challenged mice had an average total pathology score of approximately 4 (Figure 5B). Mice vaccinated with wt RSV showed significant protection relative to mock-vaccinated mice. This is in agreement with our previous work [32,82], in which we also noted that interstitial pneumonia and edema scores correlated well with overall vaccine effectiveness [32]. These two disease parameters, as well as neutrophils and mucus, had a score of 0 for wt RSV-vaccinated challenged mice (Figure 5C). In contrast, none of the VLP amounts protected mice from pathology, and the 25 µg group showed a significantly higher pathology score than mock-vaccinated mice. This was unexpected as the 25 µg VLP-GCR group showed less weight loss than the other VLP groups. The high pathology observed in the 25 µg group was mostly driven by relatively high scores for interstitial pneumonia and edema (Figure 5C). The 50 µg and 100 µg VLP-GCR groups, which displayed the most severe weight loss, showed less interstitial pneumonia than the 25 µg group and low edema scores, but they were the only vaccine groups in which mucus was detected (in 2 of 5 mice). Thus, the severe weight loss in the 50 and 100 µg groups appeared to be less driven by interstitial pneumonia and edema, but possibly by disease mechanisms related to mucus formation.

### 3.6. A VLP Expressing Prefusion F Induces Both Anti-F and Anti-N Abs after IN Vaccination (Figure 6)

The successful induction of anti-N Abs by VLP-GCR suggested that failure to induce anti-G Abs was specific to the GCR peptide and not due to the VLP itself or the IN administration method. To confirm this, we similarly generated a VLP that carries the preF protein instead of the hybrid Fstem GCR protein (termed VLP-preF). The preF gene we used has been previously described [32] and contained the four DS-Cav1-based mutations to stabilize the prefusion conformation. In addition to the ectodomain mutations, we also mutated the final two cytoplasmic tail amino acids to alanine (S573A and N574A), as described in Section 3.1 for GCR-137-211-AA. VLPs were produced in 293 cells and semi-purified, as above, and characterized via Western blotting (Figure 6A), as described in Section 2 (to detect F, motavizumab was used). Also, to detect N, we used the flag-tagged N protein mentioned in Section 3.4. VLP-preF was used to vaccinate mice to ask whether they were capable of inducing anti-F Abs. Animals were thrice vaccinated IN, as in Section 3.4, with two different doses of VLP-preF (25 and 50 µg). Similar to our observations with VLP-GCR, we found that vaccination with VLP-preF was relatively well-tolerated, with only minor weight losses occurring after vaccinations in all groups, including the mock-vaccinated group, and most weight loss occurring after wt RSV vaccination (Figure S5). 

Serum Ab levels were again compared to those of wt RSV or mock-vaccinated mice via ELISA, in which plates were coated with purified preF, postF, or N antigens (Figure 6B,C). Both VLP-preF doses induced substantial levels of anti-preF Abs, with the levels induced by 25 μg VLP-preF equal to those of mice similarly vaccinated with wt RSV. We also tested the anti-postF Ab levels. As observed previously with a live virus expressing preF [32], the preF antigen increased the proportion of anti-preF Abs in the anti-F Ab population. This was best evidenced in the 25 μg VLP-preF group, which, compared to wt RSV vaccination, induced similar levels of preF Abs but significantly lower levels of anti-postF Abs (Figure 6B,C, right panels). Similar to VLP-GCR, VLP-preF induced anti-N Ab levels which were significantly lower than the levels induced by wt RSV but were nevertheless substantial (Figure 6D). Thus, both VLPs induced anti-N Abs, and preF was much more immunogenic than the Fstem–GCR hybrid protein under the same vaccination conditions. Together, these findings indicate that authentic RSV VLPs in combination with IN administration (without added adjuvant) can effectively induce anti-viral Abs and (in case VLP-preF) a preF-focused response, and they confirm that the poor immunogenicity of the GCR is the most likely cause of low anti-G Ab levels in VLP-GCR vaccinated mice.

### 3.7. VLP-preF Vaccination Does Not Protect from Lung Pathology after Challenge in Spite of the Presence of Anti-preF Abs (Figure 7)

Mice vaccinated in Section 3.6 were challenged with 2 million PFU of wt RSV four weeks after the final boost. Mice were weighed daily and were sacrificed on day 5 post-challenge to examine lung pathology. Lungs were processed for H&E staining and scored blindly by an ACVP-certified pathologist, as described in Section 3.5. As expected, and as observed in Figure 5, wt RSV-vaccinated mice rapidly re-gained their initial weights (Figure 7A). For VLP-preF vaccinated mice, the weight loss pattern was similar (25 μg group) or higher (50 μg group) than that of mock-vaccinated mice. By day 5 post-challenge, the latter had lost ~20% of their initial weights. In spite of the presence of high levels of anti-preF serum Abs (Figure 6B), mice vaccinated with VLP-preF showed total pathology scores similar to those of mock-vaccinated mice, whereas wt RSV-vaccinated mice were protected (Figure 7B). Individual parameter scores were also similar between VLP-preF and mock-vaccinated mice, with the exception that mucus was detected predominantly in the VLP-preF vaccinated mice.

Thus, the challenge-induced weight losses observed in VLP-GCR- and VLP-preF-vaccinated animals were higher than in wt RSV-vaccinated and challenged mice and resulted in lung pathology similar (or higher in case of 25 μg VLP-GCR) to that of mock-vaccinated mice. Figure 5 and Figure 7 suggest that the enhanced pathology and high weight losses may be related to an increase in mucus levels. VLP-GCR and VLP-preF, therefore, do not constitute good stand-alone vaccines for the RSV-naive population.

## 4. Discussion

### 4.1. Rationale and Design of VLP-GCR

In a recent paper, it was shown that anti-G Abs blocked infection of HAE cells in the absence of complement [57], underscoring the potential of G to help protect against RSV. Over the years, ample data have been provided that show or suggest that focussing anti-G immunity on the G protein CCR can be further beneficial for vaccine-induced protection against RSV [16,17,46,47,48,49,50,51,52,53,55,57,60,61,85]. However, for reasons unknown, the G CCR appears poorly immunogenic, which is also true in the context of the full G protein [58,59]. Other groups have published vaccine studies in which the G CCR was presented (without its flanking highly glycosylated variable domains) in live [60], VLP [61], and micro or nanoparticle [17,53,85,86] approaches (for review, see [54]). In the micro and nanoparticle approaches, the G CCR induced anti-G Abs that contributed to protection. In the VLP approach, presentation of the G CCR combined with F offered better protection than full G combined with F [61]. 

In Rainho-Tomko et al. [17], the CCR was multi-merized via a foldon domain and used as a nanoparticle vaccine that included a prefusion-stabilized F. When delivered IM, high anti-CCR Ab levels were induced in macaques, as well as high levels of RSV-neutralizing Abs, and mice were protected from challenge without adverse effects. 

For this study, we designed VLP-GCR, a VLP that presents only G amino acids 137-211 (which includes the G CCR and the HBD), while otherwise mimicking a wt filamentous RSV particle. This was achieved by grafting the GCR onto Fstem, a domain that was shown by our lab and promises to be essential for, and to play a role in, RSV assembly. The Fstem/GCR hybrid construct was screened for surface expression and recognition by known anti-CCR Abs to ensure effective presentation. An advantage of using a homologous packaging system is that the VLPs include additional viral antigens (N, P, M), which, in our case, and in general, are well conserved. The presence of multiple viral antigens in one VLP vaccine has potential to broaden the anti-RSV immune response, thereby improving cross-protection and reducing the formation of escape mutants. Although we previously showed that N is not required for VLP assembly [18], we opted to include N because humans mount a significant humoral and cellular response to N, and N is increasingly recognized as a virulence factor [21,22,23,24,25,27,29,65]. Whereas several studies show that G and F antigens are ultimately better when combined in one vaccine [15,87], this study served to test our approach and GCR antigen design and its ability to raise protective Abs. In contrast to most other subunit approaches, we applied our VLPs IN because the initial site of infection is a factor in the induction of tissue-resident memory cells and because of the mounting evidence for the importance of local mucosal immunity in reducing RSV infection and disease severity in animals and humans [29,68,69,70,71]. The study was conducted without adjuvants for the reasons stated in the introduction.

### 4.2. VLP-GCR Induces Very Low Levels of Anti-G Abs

VLP-GCR incorporated high levels of the GCR-presenting hybrid protein Fstem/GCR137-211-AA and resembled a filamentous wt RSV particle via SEM. Vaccinations with VLP-GCR were well tolerated in mice. However, very low levels of anti-G Abs were induced even after three vaccinations with VLP-GCR and high dosages. In contrast, a VLP similarly generated but presenting preF instead of G induced high levels of anti-F Abs. Both VLP-GCR and VLP-preF induced high levels of anti-N Abs. These results show that our VLPs were immunogenic after IN administration without an adjuvant, but they also indicate that our GCR antigen was poorly immunogenic in spite of strong recognition by known anti-GCR Abs L9 and 131-2G and the absence of the majority of the variable glycosylated regions. Since preF and N Abs were induced, poor immunogenicity of the GCR is likely a quality inherent in the GCR construct. It is possible that the addition of an adjuvant could render our GCR construct more immunogenic, but we have not explored this option due to safety concerns in humans with IN-applied adjuvants [72,73,74,75]. Since the G protein central region contains an important receptor-binding domain (CX3C), it is possible that this region has evolved to be poorly immunogenic to avoid immune recognition in vivo. On Western blots, Fstem/GCR had a smeary appearance, indicating a range of molecular weights. Thus, Fstem/GCR maintains some glycosylation, and it is possible that the attached carbohydrates protect the GCR.

### 4.3. Vaccination with VLP-GCR and VLP-preF Fail to Protect, and VLP-GCR Vaccination Results in Severe Weight Loss and Pathology after Challenge

It was previously shown that G protein is not necessary for enhanced disease to occur [88], and G does not always induce enhanced disease [15,16,17,85]. In our case, VLP-GCR induced very low levels of anti-G Abs, and challenge of vaccinated animals with wt RSV displayed weight losses that exceeded the post-challenge weight losses observed in mock-vaccinated mice. All VLP-vaccinated mice also displayed higher lung pathology, though this was significant only in one of the VLP-GCR groups. However, both VLPs clearly did not protect the animals, in contrast to vaccination with wt RSV. Given the low level of anti-G Abs, we had anticipated suboptimal protection by VLP-GCR, although we could not predict the impact of the presence of anti-N, P, or M immunity on lung pathology. In the case of VLP-preF, the lower dose induced anti-preF Ab levels as high as those induced by a wt RSV. Therefore, we did expect a certain level of protection from lung pathology, as the same preF antigen, when included in a live-attenuated vaccine, protected animals as well as vaccination with wt RSV [32]; this was also true in the presence of low anti-G Ab levels. It is not clear why the VLP-induced preF Abs did not protect from lung pathology. In the paragraph below, we list a few conditions previously reported to play a role in G-mediated disease and which may have contributed to excessive weight loss or failure to protect.

Previous publications have used the G protein or the G CCR in different non-live approaches and demonstrated protection or partial protection without adverse effects [15,16,17,85,89]. In contrast, other previous publications reported enhanced pathology after non-live vaccines with G protein [87,90,91,92,93]. In a review by Acosta et al. [94], a range of potential causes of severe pathology were reported, including increased IL-4, IL-5, and IL-13, exacerbated Th17 responses, eosinophils, aberrant T cell trafficking, Th2 bias, suppression of Treg activity, poor TLR signalling, immune complex disposition, low IFN type I and III levels, and high levels of pro-inflammatory cytokines. Recently, a role for IL-17 (in conjunction with IL-23) in RSV disease has been further investigated. It was discovered that in the most severely ill patients, very high levels of IL-17 and mucus were present, as well as low levels of interferons and CXCL10 [95,96]. It is thought that high IL-17 expression induces (mostly pro-inflammatory) cytokine and chemokine secretion by different cell types, including epithelial cells, endothelial cells, and macrophages. These cytokines then recruit and activate different immune cells, ultimately leading to high levels of inflammation and mucus expression [96,97,98,99]. A limitation of our study is that we did not examine the lung cytokine milieu, and therefore we do not know whether VLP-GCR induced a similar chain of events as described above for IL-17. Because enhanced disease-like responses have been described for other subunit type vaccines, including G-based vaccines, we think it is possible that VLP-GCR induced Th2 and Th17 responses that ultimately increased mucus expression and weight loss. However, the role of IL-17 is complex, and IL-17 can also be important in ‘normal’ disease resolution [95,96,100]. We did examine the lung pathology to look for potential causes of the high weight loss observed after challenge of VLP-vaccinated mice. Note that eosinophils were later shown to not play an important role in VED [101] and did not appear to impact pathology in our previous studies [32] and hence were not included in our scores. Out of all disease parameters, mucus was the parameter that showed the biggest differences between VLP-vaccinated and mock-vaccinated mice. The latter did not result in noticeable mucus levels, as is typically observed with RSV strain A2 [102]. In our case, this implicates mucus as a potential cause for enhanced weight loss. A more severe, mucogenic disease, including high levels of IL-13, has been described for animals infected with RSV A2-line19F [102,103]. The contributions of Th17 cells and IL-17 were not examined in the latter studies but presumably play a role in the observed severe pathology. For RSV A2-line19F specifically, F protein residues 357 and 371 were involved in mucus formation, but the underlying causes remain unclear. Our data suggest that preF and GCR could each play a role in mucogenic responses. The G protein, specifically, was previously found to induce excessive levels of mucus after challenge with RSV A2-line19F [87]. In our case, the GCR-displaying VLPs with the most weight loss also induced mucus, suggesting a role for the GCR in mucogenic pathology. Alternatively, other factors common to VLP-GCR and VLP-preF may contribute, such as the viral N, P, or M proteins, or even host proteins, incorporated into the VLPs.

### 4.4. In Summary

We screened a number of hybrid proteins to express and present the central region of G and generated RSV-based VLPs. Animals were vaccinated IN with the intention to closely mimic an infectious RSV particle and the natural route of RSV entry. These VLPs also included three core antigens, against which humoral and cellular immunity have been demonstrated in humans, and which have potential to broaden the response. Comparison of a VLP expressing the central region of G or the preF protein revealed that both VLPs were immunogenic when applied IN without adjuvant. However, the GCR appeared significantly less immunogenic than preF under the same vaccination conditions. Both VLPs caused equal or more challenge-induced weight loss and lung pathology than mock-vaccinated mice and also induced more mucus in the lungs. Excessive mucus can contribute to severe RSV disease, and it appears that the VLPs induced a more mucogenic response than mock-vaccination. However, it remains unclear as to what underlies the increased disease induced by our VLPs. Independent of the cause of the observed weight loss and increased pathology, it is clear that neither VLP is suitable for the vaccination of RSV-naive individuals. However, VLP-preF might be suitable to serve as a booster vaccine in RSV-experienced individuals, in which subunit vaccines generally do not have adverse effects upon infection or challenge with wt RSV [76,90,91]. As a booster vaccine, IN-applied VLP-preF could enhance the immune response to multiple RSV antigens, direct the anti-F Ab population towards preF, and induce both lung-resident and systemic immunity.

## Data Availability

The original contributions presented in the study are included in the article/Supplementary Material, further inquiries can be directed to the corresponding author.

## Supplementary Materials

The following supporting information can be downloaded at: https://www.mdpi.com/article/10.3390/v16060843/s1, Figure S1: Negative control sample for high resolution analysis of VLP-GCR; Figure S2: Antiviral Abs induced after vaccination with VLP-GCR; Figure S3: Relative weight changes after vaccination with VLP-GCR; Figure S4: Validation of anti-nucleoprotein (N) ELISA; Figure S5: Relative weight changes after vaccination with VLP-preF.
