# Supplementary material for: Intranasal Vaccination with a Respiratory-Syncytial-Virus-Based Virus-like Particle Displaying the G Protein Conserved Region Induces Severe Weight Loss and Pathology upon Challenge with Wildtype Respiratory Syncytial Virus
Source: Viruses. 2024 May 24;16(6):843. doi: 10.3390/v16060843 (PMC11209524; doi:10.3390/v16060843)
Supplement: Supplementary file 1 [file viruses-16-00843-s001.zip › viruses-2985248-supplementary.pdf]

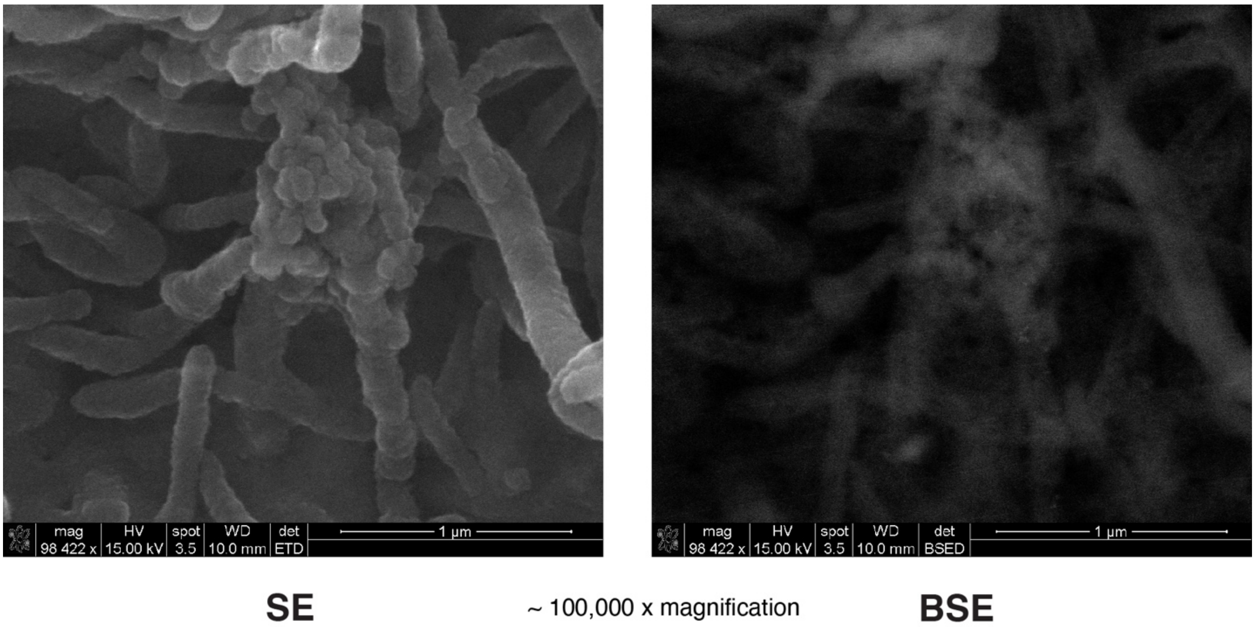

Figure S1. Negative control sample for high resolution analysis of VLP-GCR. HEp-2 cells grown on plastic coverslips were transfected with plasmids expressing N, P, M, and a hybrid Fstem protein containing GCR137-211-AA. At 26 hpt, the GCR was labeled with a non-relevant primary Ab (AcV5 [83]) followed by a secondary Ab conjugated to 10 nm gold, washed, and processed for SEM. Samples were examined using secondary electron (SE)(left panel) and backscattered electron (BSE) (right panel) modes, and photographed at 100,000x magnification.

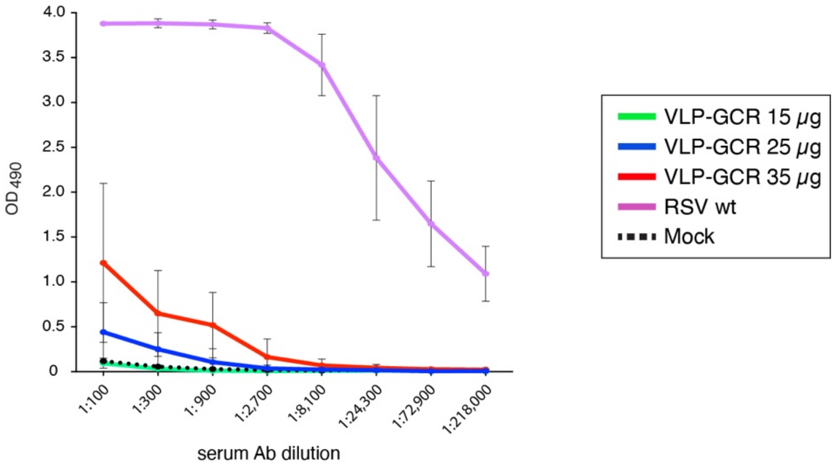

Figure S2. Antiviral Abs induced after vaccination with VLP-GCR. Different amounts of VLP-GCR (15, 25, or 35 µg) were used to vaccinate 8-week old BALB/c mice (n=5/group). This was done IN without adjuvant (three vaccinations as shown in Fig. 4B). As a comparison, mice were similarly vaccinated with wt RSV, or mock (PBS) vaccinated. Three weeks after the final vaccination, blood samples were taken and anti-G Ab levels were determined by ELISA, using commercially acquired purified G protein (Sino Biological) to coat the plates. Error bars represent the standard deviation of the mean of 5 individual mice.

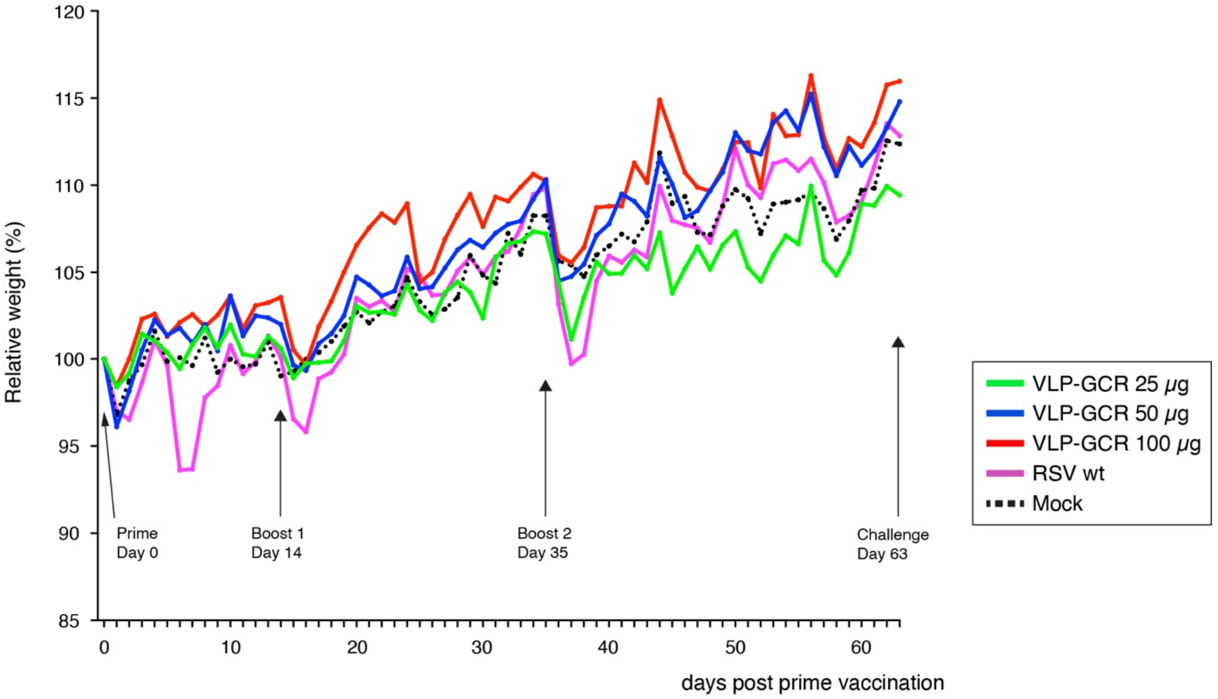

Figure S3. Relative weight changes of animals after vaccination with VLP-GCR. BALB/c mice (n=5/group) were thrice vaccinated with VLP-GCR (boosts given two weeks and five weeks post prime) with 25, 50, or 100 µg. Vaccinations were done IN without adjuvant. Mice were weighed daily. Weights are shown relative to the weight on day 0. Times of prime, boost, and challenge vaccinations are indicated by arrows.

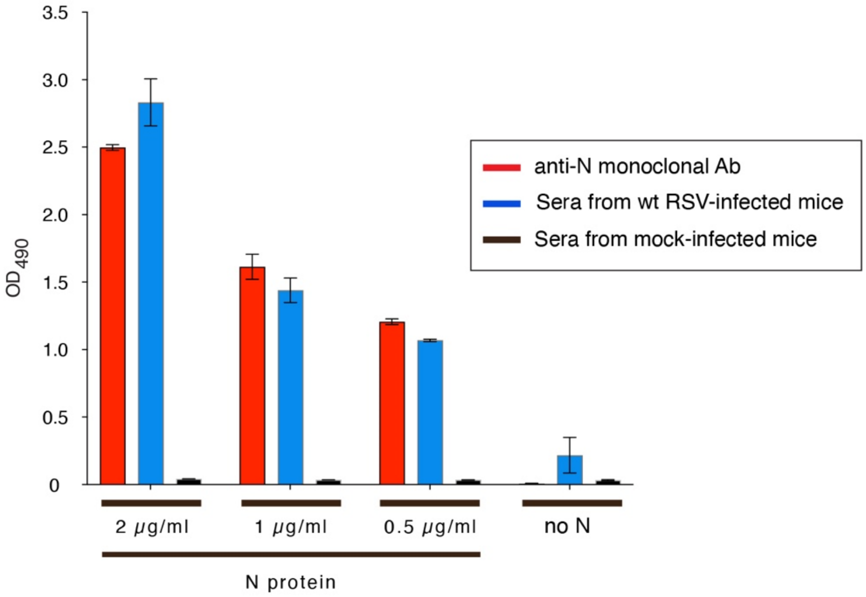

Figure S4. Validation of anti-nucleoprotein (N) ELISA. Different amounts of purified his-tagged N protein (Sino Biological) were incubated on nickel-coated plates overnight. Plates were blocked, washed, and then incubated with the following primary Abs: Anti-N monoclonal Ab (AbD serotec), pooled sera from mice (n=5) twice infected with wt RSV, or pooled sera from mock-infected mice (n=5). After primary Abs, plates were processed for standard ELISA. Error bars represent the standard deviation of the mean of triplicate samples (technical replicates of pooled sera).

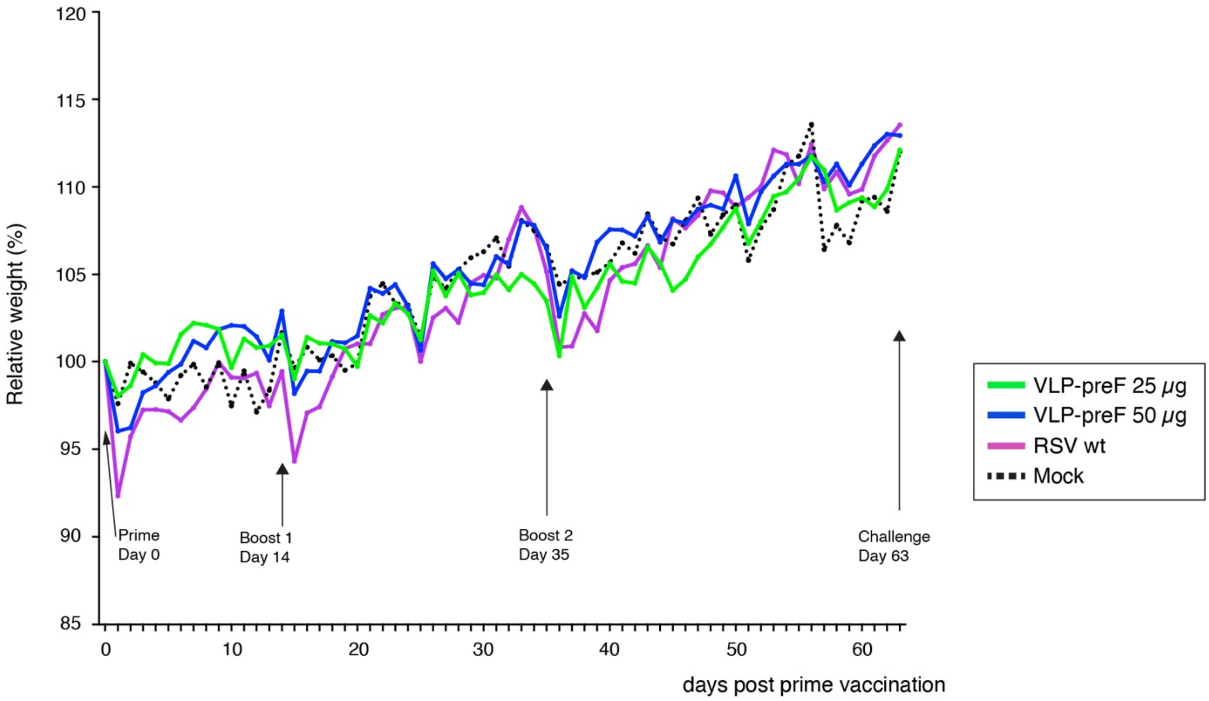

Figure S5. Relative weight changes of animals after vaccination with VLP-preF. BALB/c mice (n=5/group) were thrice vaccinated with VLP-preF (boosts given two weeks and five weeks post prime) with 25 or 50 µg. Vaccinations were done IN without adjuvant. Mice were weighed daily. Weights are shown relative to the weight on day 0. Times of prime, boost, and challenge vaccinations are indicated by arrows.
